# Assessing the Micro- and Macroscopic Changes of Chemically Altered Human Bone and Teeth

**DOI:** 10.3390/biom16010001

**Published:** 2025-12-19

**Authors:** Shelby R. Feirstein, Maria J. Castagnola, Dakota M. Bell, Mayaas Hassan, Alixs M. Pujols, Luis L. Cabo, Joe Adserias-Garriga, Sara C. Zapico

**Affiliations:** 1 Department of Applied Forensic Science, Mercyhurst University, Erie, PA 16546, USA; bell.dakota19@gmail.com (D.M.B.); lcabo@mercyhurst.edu (L.L.C.); jadseriasgarriga@mercyhurst.edu (J.A.-G.); 2 Department of Chemistry and Environmental Science, New Jersey Institute of Technology, Newark, NJ 07102, USA; mc997@njit.edu (M.J.C.); mayaaskhassan@gmail.com (M.H.); alixs1699@hotmail.com (A.M.P.); 3 Anthropology Department and Laboratories of Analytical Biology, National Museum of Natural History, Smithsonian Institution, Washington, DC 20560, USA

**Keywords:** forensic sciences, forensic anthropology, molecular genetics, DNA, STR, sodium hydroxide, hydrochloric acid, taphonomy, personal identification

## Abstract

Sodium hydroxide (NaOH) and hydrochloric acid (HCl) are household chemicals used to disfigure victims in forensic contexts due to their high availability and apparent effects, which alter both the structural integrity and composition of skeletal elements. NaOH dissolves soft tissues and produces violent, exothermic reactions but, ostensibly, fails to alter the structure and color of bones and teeth. HCl is considered one of the most destructive chemical agents utilized, causing rapid demineralization of hard tissues. Current works focus on total dissolution times, rather than on discrete changes and the potential for personal identification. This research aims to comprehensively assess the intervallic micro- and macroscopic changes occurring in chemically altered bones and teeth. Analyses were conducted to investigate how morphological shape and surface area-to-volume ratios may affect the degree of alteration and to evaluate the feasibility of DNA isolation and profiling. The relationships between these factors were not linear, and the results show a variable pattern of alteration and DNA yields depending on the treatment and duration of exposure. Teeth were found to be better sources for obtaining higher quality and yield of DNA compared to bones, and complete STR profiles were obtained from all tooth samples. Overall, this pilot study highlights the challenges of analyzing taphonomically altered remains and underscores the need for effective identification methods.

## 1. Introduction

Identification is an inherent human right that medico-legal professionals aim to provide to individuals posthumously. More specifically, forensic anthropologists help in returning identity to the deceased. However, a variety of taphonomic agents can affect remains, making it more challenging to identify the decedent. Exposure to household corrosive (acidic) and caustic (alkali) substances changes both the structural integrity and composition of skeletal elements as hydroxyapatite crystals and DNA become chemically modified by the pH of the depositional environment [1,2]. Consequently, the possibility of DNA recovery and characterization after chemical alteration is largely unknown.

However, DNA exhibits substantially greater preservation in hard tissues, like bone and teeth, compared to soft tissues, because DNA chemically binds to hydroxyapatite that then provides stability and protection to DNA molecules [2,3]. When DNA becomes exposed to environmental conditions, the nuclear DNA (nDNA), which begins to break down naturally after death, becomes highly susceptible to environmental degradation [4]. Antemortem predispositions (i.e., age or pathology-related factors) also contribute markedly to the integrity of skeletal material at death. Compromised nDNA may inhibit profiling due to limited yields, higher allelic dropout, and a greater likelihood of contamination [4]. Certain conditions, such as exposure to chemicals, can significantly affect the preservation potential of DNA, resulting in reduced yields in biological tissues.

Hydrochloric acid (HCl), a strong acid, is sold commercially as muriatic acid and effectively cleans through decalcification, which causes the demineralization of calcium phosphate (carbonated hydroxyapatite) in bone and teeth [5,6,7]. Given that neither is uniformly mineralized, it is expected that areas would become chemically etched at varying rates as carbonate is released. That is, more carbonate increases acid solubility and dissolution rates [8]. With prolonged exposure, bony and dental structures are stripped down to their flexible collagen matrices and eventually reach complete dissolution [5].

Sodium hydroxide (NaOH), a strong base, effectively cleans via saponification, which attacks fatty substances, like bone marrow or dental pulp, and damages collagen over time [7,9,10]. Collagen damage leads to changes in structural organization that end with gelatinization [11]. The violent exothermic reaction, emitted when NaOH contacts water, would likely increase the rate of chemical attack on skeletal structures, as collagen is already particularly sensitive to alkali environments [11].

In most taphonomic conditions, teeth are found to preserve better than bones, given the different tissue composition, multi-layer protection of the soft tissue, presence of crystalline hard tissue structures, and location within alveolar bone [12]. However, some studies show that this structural protection is limited when exposed to strong acids [6]. Bone, on the other hand, maintains relative resistance to degradation, but is more susceptible due to dehydration of “wet” bone and collagen degradation, which subsequently affects the inorganic hydroxyapatite structures [13]. Knowing this, sample selection becomes increasingly important, especially for human remains found in complicated taphonomic conditions.

The current recommendations for DNA sampling favor cortically dense, weight-bearing bones—such as the femur—and teeth [3,14]; however, these elements are not always available for sampling and subsequent analyses. Sample selection for genetic profiling may also disrupt anthropological analyses when samples are excised or taken in their entirety for such testing. Researchers have hypothesized that small trabecular elements, like those in hands and feet, could be richer sources of DNA, compared to cortical bone, given the proportion of DNA yield per unit mass [3,14,15,16]. This same principle applies to teeth with the largest pulp volume and root surface area, such as molars, which can better withstand degenerative changes in DNA over time [17]. Most importantly, a key consideration is that the shape, size, composition, and function of individual bones contribute collectively to an element’s preservation potential, which is defined here as elemental resistance to taphonomic alteration and molecular degradation [18,19].

The first phase of this pilot study aims to comprehensively assess micro- and macroscopic changes in fully skeletonized remains exposed to household chemicals for up to 2 h. Analyses were conducted to test how bone shape and surface area-to-volume (SFA:VOL) ratios may affect the degree of alteration and evaluate the possibility of DNA isolation and profiling. The second phase of this experiment was then conducted using teeth, which are often considered better sources for obtaining higher-quality yield of DNA compared to bones [12,20].

This study aims to assess how dimensional changes in bones and teeth, altered by household chemicals, will affect the potential for DNA extraction.

## 2. Materials and Methods

### 2.1. Sample Selection and Data Collection

Twenty-three total carpals, metacarpals, and phalanges, from one donated, middle-aged, biologically male individual were generously provided by Mercyhurst University’s Ted A. Rathbun Osteological Collection for experimentation. The previously fleshed, fully articulated hand and three disarticulated right-hand elements (two distal manual phalanges (DMP; ray 1 and 3) and one proximal manual phalanx (PMP; ray 1)) were frozen for nine months before processing and experimentation. The remains were then macerated using field-approved methods (crockpot, warm water, laundry detergent (1 tbsp), and Tergazyme enzymes (4 tbsp) (Alconox, Inc., White Plains, NY, USA)) [21].

**Figure 1:**
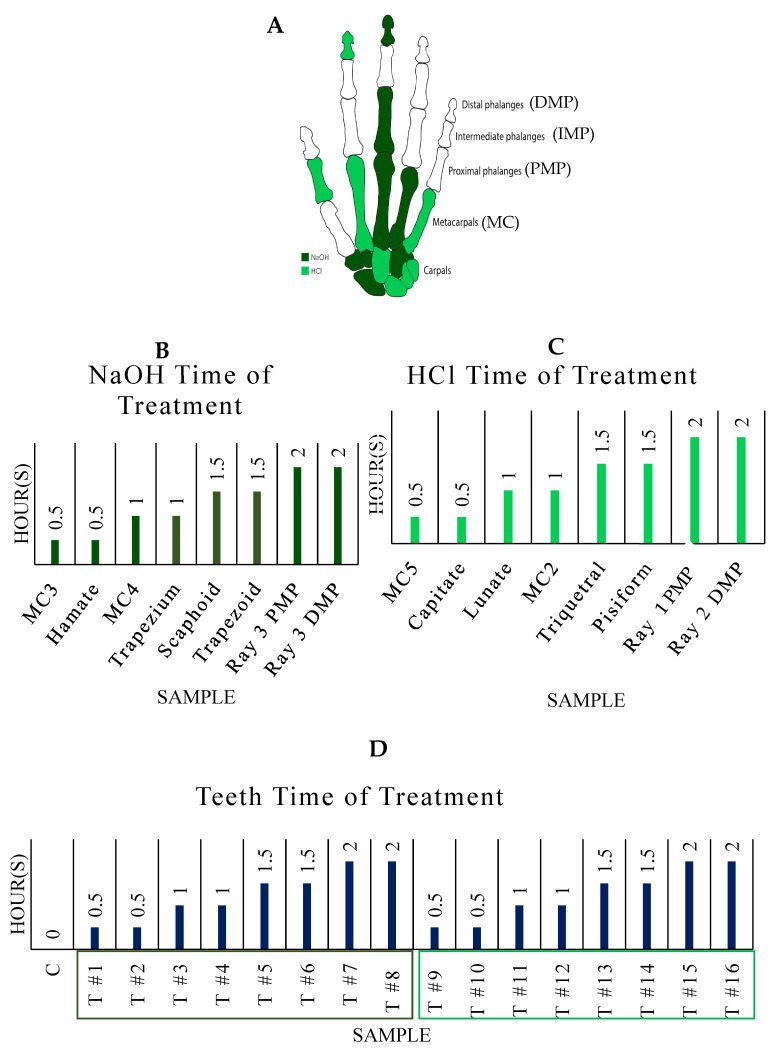
Overall view, in anatomical position, of left-hand bone samples used in respective treatments (**A**); NaOH samples and their given time interval (**B**); HCl samples and their given time interval (**C**); Molar samples and their given time interval (**D**). NOTE: DMP: distal manual phalanx; IMP = intermediate manual phalanx; PMP = proximal manual phalanx; MC = metacarpal.

**Figure 2:**
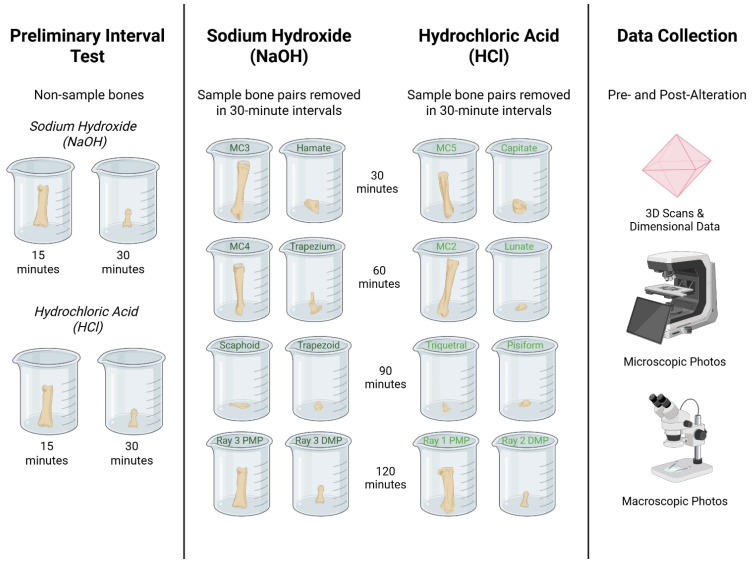
Schematic representation of the experimental design, including the preliminary interval test of non-sample bones, bone pairings in their respective treatments (NaOH or HCl), duration of treatment (interval), and the pre- and post-alteration data collection steps. For further details, refer to the materials and methods. Created in BioRender. Feirstein, S. (2025) https://BioRender.com/ljnqy7w (accessed on 20 November 2025).

Sixteen left-hand elements were exposed to NaOH and HCl (Figure 1), eight elements per treatment and two per interval (see Figure 2). Four additional left-hand elements, one DMP, one intermediate manual phalanx (IMP), and two PMPs were used for preliminary interval tests before experimentation. The right ray 1 PMP was used as the control. The remaining skeletal elements were not used for experimentation.

Pre- and post-treatment length, width, volume (VOL; mm^3^), and surface area (SFA; mm^2^) data were recorded using the Artec Space Spider handheld 3D scanner (Artec 3D, Santa Clara, CA, USA) (0.05 mm 3D point accuracy and 0.1 mm 3D resolution), and bone weight was measured to the closest ± 0.01 g for all sample and control elements. The 3D scanner was calibrated using the system’s internal diagnostic protocol. All individual scan frames were below the 0.3 threshold, the acceptable resolution range, before fusing the 3D mesh models. Surface area and volume were then captured using the measurement function in Artec Studio 16 (version 16.0.8.2) (Artec 3D, Santa Clara, CA, USA)*.* The length and width measurements were extracted after setting a custom orthogonal plane, a reference plane for precise positioning of the object of interest (i.e., the bone model), thereby increasing measurement accuracy and quality control. Measurements of tubular bones captured the medial/lateral width of the base and head, as well as the length of the bone at midline. Two reference points were placed on the medial and lateral aspects of the tubular bones and one on the head. Similarly, two reference points were selected on the medial and lateral aspects of the carpal bases and one on the most superior aspect. Measurements of round bones captured the medial/lateral width, the superior/inferior length, and the width of the most prominent base or facet.

Bones were examined and photographed with an Echo Revolve inversion microscope (1.25× lens and Brightfield transmitted light setting) (Echo, San Diego, CA, USA) and an Olympus *SZX16* whole specimen microscope (visible within the Olympus CellSens Dimensions software Version 4.3) (Evident Scientific, Waltham, MA, USA) .

Predefined bone pairs were determined using a randomized block design of the pre-alteration data in Excel (NaOH: Hamate + Metacarpal 3; Trapezium + Metacarpal 4; Scaphoid + Trapezoid; Ray 3 PMP + Ray 3 DMP and HCl: Capitate + Metacarpal 5; Lunate + Metacarpal 2; Triquetral + Pisiform; Ray 1 PMP + Ray 2 DMP). The pairings were created to test the effects of each environment on round and tubular bones, followed by pairings with the most similar volume measurements to avoid significant gaps in the data (Figure 2).

The second phase of the experiment utilized nine adult third molars acquired from private dental clinics (Figure 1). Teeth were surgically removed from biologically female individuals (age range: 18–48; mean: 31.0 ± 9.2 years; mode: 28 years old). Seven donors were between 18 and 30 years old, and two were between 45 and 50 years old, including the unaltered control sample. The teeth were weighed to the nearest ± 0.01 g, paired by weight, and examined and photographed using the aforementioned whole-specimen microscope. Data collection in this phase was focused on macroscopic changes and the viability of DNA analysis. Future studies will capture additional dimensional data for comparative value against bone.

### 2.2. Experimental Design

Preliminary ‘test’ elements (PMPs, IMP, and DMP) were used to determine the duration (15 versus 30 min) of experimental intervals and were not tested further, and no additional data were collected (Figure 2).

The chemical alteration protocol consisted of four intervals (total: 2 h), with bone pairs being pulled from their respective solutions (NaOH or HCl) every 30 min. Instant Power^®^ Crystal Lye Drain Opener (Instant Power Corporation, Dallas, TX, USA) was mixed at a 1:1 ratio (~25 M) of 100 g NaOH:100 mL H_2_O in each plastic beaker (8 total). 100 mL of undiluted TransChem Muriatic Acid (31.45%) was added to an additional eight beakers (Harcros Chemicals Inc., Kansas City, MO). The pH was tested before experimentation and after bone removal at each interval using Hydrion pH 1.0–2.0 strips (MicroEssential Lab, Brooklyn, NY, USA) (Figure 2).

### 2.3. Sample Preparation and DNA Extraction of Bone

An autopsy saw and bench vise clamp were cleaned with 99.9+% isopropyl alcohol and 10% bleach between each cut. The bones were stabilized in the clamp, then cut in half (carpals) or cut into cross-sections (metacarpals or phalanges). Due to the size of the elements, no separation between cortical and trabecular bone was performed.

The samples were crushed into a fine bone powder in mortars and pestles, which were sterilized with isopropyl alcohol and irradiated under UV light for 15 min between each sample. Samples were weighed out to approximately 0.126 g, which was determined to be the lowest quantity available across all samples. The DNA extraction protocol was completed using the Qiagen DNeasy Blood and Tissue Kit (Qiagen, Germantown, MD, USA) and a Thermo Scientific Precision GP05 (Thermo Fisher Scientific, Waltham, MA, USA) for water bath incubation.

### 2.4. Sample Preparation and DNA Extraction of Teeth

Cementum and enamel were removed with a diamond dental bur to expose the dentin. Dentin was cut along the midline using a diamond dental disc, and the pulp was removed with a spoon excavator. The dentin was then isolated and ground with an agate mortar and pestle before being aliquoted into 200 mg each [22,23,24]. The same extraction and quantification protocols for the bone were then applied to the teeth. This protocol for obtaining DNA from dentin and pulp ensures a higher quantity and quality of DNA, as dentin and pulp are protected from chemical insults by cementum and enamel, as previously described [23,24].

### 2.5. DNA Quantification and Quality Assessment

An initial quantification was performed using a NanoDrop spectrophotometer (Thermo Fisher Scientific) to assess whether DNA was present in post-alteration samples. The samples were stored at −80 °C.

Per FBI guidelines, human-specific quantification was then performed using the Promega^®^ PowerQuant^®^ System (Promega Corporation, Madison, WI, USA) on the QuantStudio (Thermo Fisher Scientific) according to the manufacturer’s protocol. The quality of the samples was assessed by determining the Degradation Index (DI) and the potential presence of inhibitors using the Internal PCR Control (IPC).

### 2.6. STR Profiling

The Promega^®^ PowerPlex^®^ Fusion 6C System (Promega Corporation) was used to amplify 23 autosomal STR markers, amelogenin, and 3 Y-STRs from 0.5 ng of DNA.

The SeqStudio Genetic Analyzer (Thermo Fisher Scientific, Waltham, MA, USA) was used to conduct Fragment Analysis (7 s injection time, 1200 V injection voltage, 1440 s run time, and 9000 V run voltage) and the final DNA profile analysis was completed using Microsatellite Analysis Software in Thermo Fisher Connect Platform (https://www.thermofisher.cn/cn/zh/home/digital-science/thermo-fisher-connect/all-analysis-modules.html, accessed on 2 November 2022) (peak detection threshold of 150 RFU (Relative Fluorescent Units)), as previously described [23,24].

These resulting profiles were then compared to the complete genetic profiles taken from the control elements.

### 2.7. Data Analysis

Statistical analyses were run using GraphPad Prism 10 (version 10.6.1) to assess normality and variance. Non-parametric tests were selected in most cases as a precaution because of the data’s low statistical power due to the necessarily small sample size. Statistical significance was determined at *p* ≤ 0.05.

Spearman’s Rank-Order Correlation was employed to test the strength of the observed relationships among metric variables. Wilcoxon Matched-Pairs Signed Rank Mean Comparison Tests were used to assess median differences using paired pre- and post-treatment group data. The Mann–Whitney U test was utilized to compare the distributions of two independent groups at a time.

## 3. Results

### 3.1. Micro- and Macroscopic Changes

**Figure 3:**
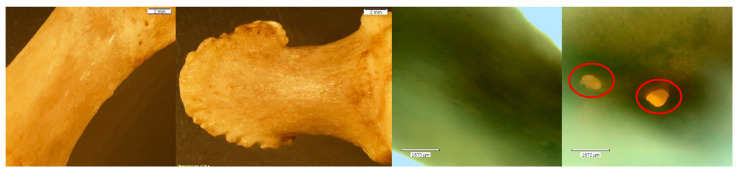
*Echo*  and *Olympus* microscope photos showing exemplars of unaltered bones of minimal porosity and of good quality. Note the uniform edges on the natural foramina of the capitate (red circles). From left to right: macroscopic view of the PMP shaft (2 mm; magnification 0.35×); macroscopic overall view of the DMP (2 mm; magnification 0.35×); microscopic view of metacarpal 5 (MC5) at midshaft (1670 μm); microscopic up-close view of the natural foramina in the capitate (1670 μm). NOTE: The PMP and DMP were later used as ‘test’ elements in the NaOH experiment and the MC5 and capitate were later treated with HCl for 30 min.

Exemplars of unaltered bone (Figure 3) are presented in contrast to both the NaOH- and HCl-altered elements. The PMP and DMP were later used as ‘test’ elements in the NaOH experiment, and the MC5 and capitate were later treated with HCl for 30 min.

**Figure 4:**
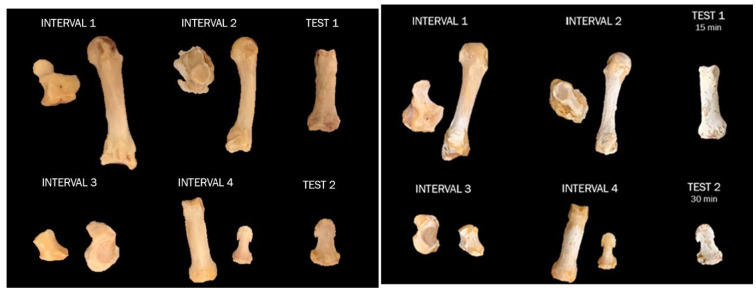
Overall view of skeletal elements prior to NaOH treatment (**left**) and after NaOH treatment for their respective amount of time (**right**). NOTE: Interval 1: 30 min; Interval 2: 60 min; Interval 3: 90 min; Interval 4: 120 min. The ‘test’ elements were used to determine the duration of experimental intervals and were not tested further, and no additional data were collected.

**Figure 5:**
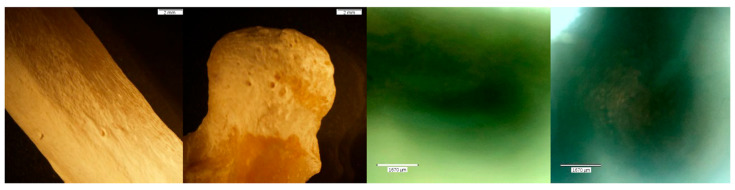
*Echo* and *Olympus* microscope photos showing bones after being submerged in NaOH for 30 min. From left to right: macroscopic view of the MC3 shaft (2 mm; magnification 0.35×); zoomed-in macroscopic view of the hamate hamulus (2 mm; magnification 0.35×); microscopic look at the midshaft of MC3 (1670 μm); microscopic view of the hamulus (1670 μm). The bones began to turn white but majorly retained their natural yellow color. The density of bone was unchanged, and no bone breakdown was visible.

**Figure 6:**
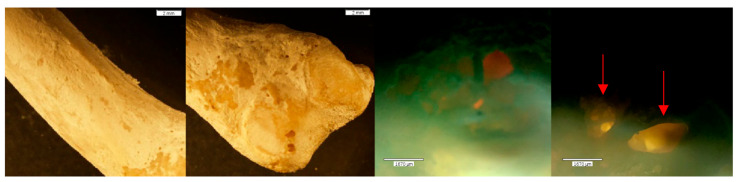
*Echo* and *Olympus* microscope photos showing bones after being submerged in NaOH for 60 min. From left to right: macroscopic view of the MC4 shaft (2 mm; magnification 0.35×); macroscopic view of the distal end of MC4 (2 mm; magnification 0.35×); microscopic view of the inferior surface of the trapezium where arthritic lipping was already present (1670 μm); microscopic view of the pre-existing trapezial foramina (1670 μm). The bones appear whiter in color as the sodium adheres to the surface. The yellow color of the natural bone appears patchy along the shaft but is still predominant on the facets seen on MC4. No additional bone breakdown was visible after alteration. The edges of the trapezial foramen (red arrows) are uniform, lacking any jagged edges characteristic of breakdown.

**Figure 7:**
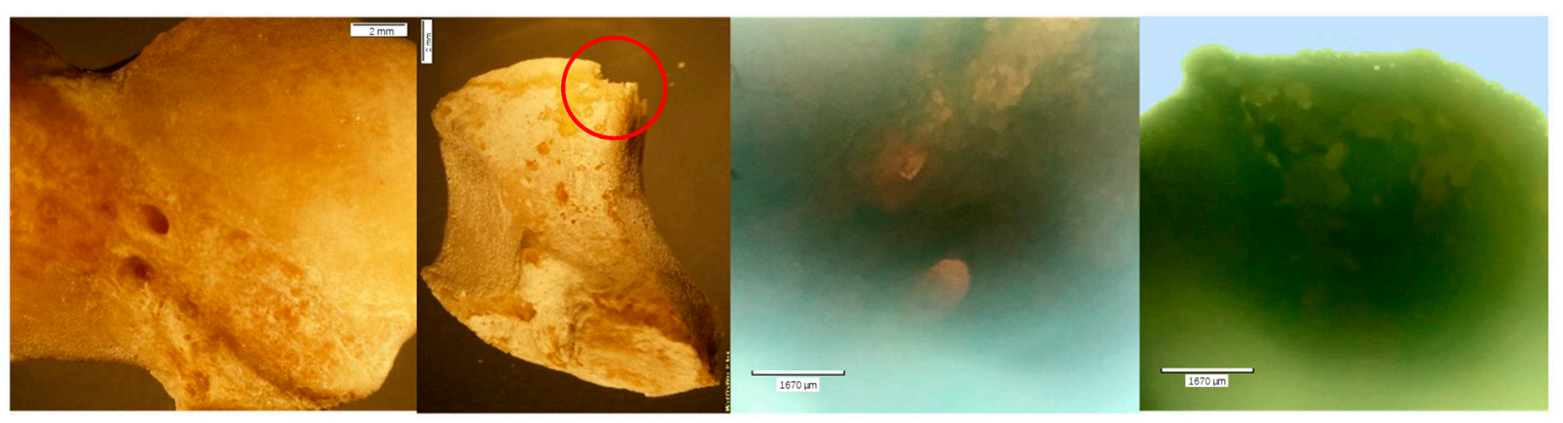
*Echo* and *Olympus* microscope photos showing bones after being submerged in NaOH for 90 min. From left to right: macroscopic view of the scaphoid body (2 mm; magnification 0.35×); overall macroscopic view of the trapezoid (2 mm; magnification 0.35×); microscopic look at the natural foramina of the scaphoid (1670 μm); microscopic view of the base of the trapezoid (1670 μm). A thin layer of white film overlaid the natural bone and the natural foramina in the body of the scaphoid remained uniform. There appears to be some sort of bone breakdown occurring on the trapezoid around the facet for the capitate (red circle). The density of the bone appears unchanged.

**Figure 8:**
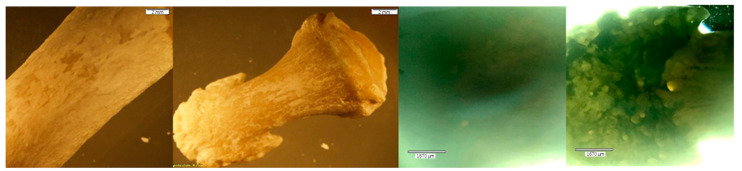
*Echo* and *Olympus* microscope photos showing bones after being submerged in NaOH for 120 min. From left to right: macroscopic view of the shaft of the PMP (2 mm; magnification 0.35×); overall macroscopic view of the DMP (2 mm; magnification 0.35×); microscopic look at the midshaft of the PMP (1670 μm); microscopic view of the head of the DMP (1670 μm). The skeletal elements appear to retain their natural density even after two hours. The white coloration, however, persists as the sodium continues to interact with the bone’s surface.

The micro- and macroscopic alterations before and after submersion in NaOH (Figure 4; overall) are shown in photographs below to best illustrate the visible physical changes. Bones soaked in NaOH showed color changes and minimal bone breakdown only after 1.5 h. While minimal changes in NaOH pH were observed between intervals 1 and 2, the pH remained stable for the remainder of the experiment (Figure 5, Figure 6, Figure 7 and Figure 8).

**Figure 9:**
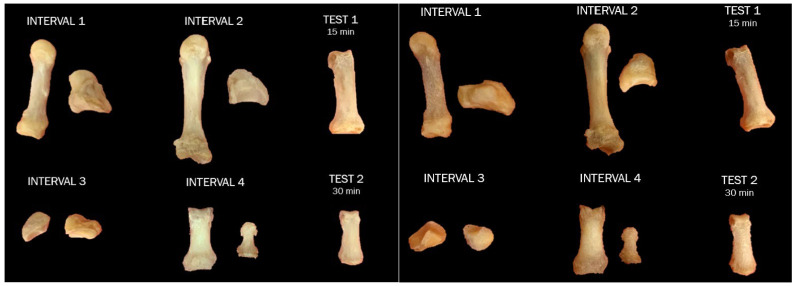
Overall view of skeletal elements prior to HCl treatment (**left**) and after HCl treatment for their respective amount of time (**right**). NOTE: Interval 1: 30 min; Interval 2: 60 min; Interval 3: 90 min; Interval 4: 120 min. The ‘test’ elements were used to determine the duration of experimental intervals and were not tested further, and no additional data were collected.

**Figure 10:**
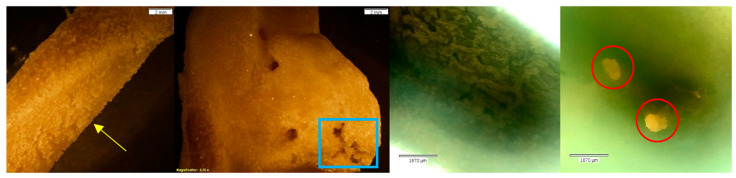
Echo and Olympus microscope photos showing bones after being submerged in HCl for 30 min. From left to right: macroscopic view of the shaft of MC5 (2 mm; magnification 0.35×); overall macroscopic view of the capitate (2 mm; magnification 0.35×); microscopic view of the midshaft of MC5 (1670 μm); microscopic view of the pre-existing foramina of the capitate (1670 μm) (see Figure 3 for comparison). The bone surface appears crystalline, and there are signs of cortical flaking (yellow arrow) and bone breakdown (blue box). The natural foramina of the capitate have irregular edges, indicative of breakdown (red circles). The density of bone, however, appears majorly unchanged.

**Figure 11:**
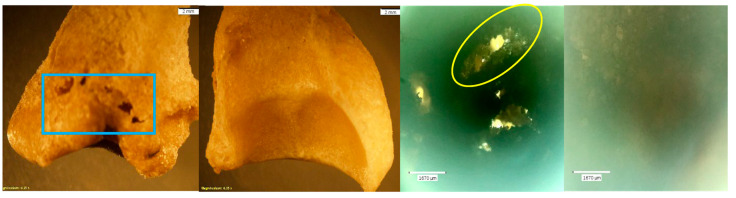
*Echo* and *Olympus* microscope photos showing bones after being submerged in HCl for 60 min. From left to right: macroscopic view of the anterior distal portion of MC2 (2 mm; magnification 0.35×); macroscopic view of the posterior portion of the lunate (2 mm; magnification 0.35×); a microscopic view of the posterior distal portion of MC2 (1670 μm); microscopic view of the posterior portion of the lunate (1670 μm). The bones appear to become increasingly vibrant in color. The surface of bone has crystallized and is shiny in appearance. The chemical ate away at the cortical bone, particularly on the distal end of MC2 (blue box). The lunate appears to have been less affected than MC2. The complete holes (as in those that extend through the base) that developed in the distal portion of the metacarpal have the appearance of melted Styrofoam (example: yellow oval). The density in the lunate appears unchanged.

**Figure 12:**
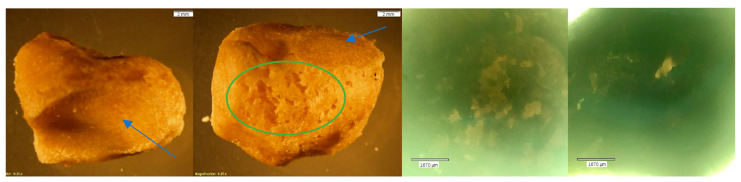
*Echo* and *Olympus* microscope photos showing bones after being submerged in HCl for 90 min. From left to right: macroscopic view of the inferior surface of the triquetral (2 mm; magnification 0.35×); overall view of the triquetral (2 mm; magnification 0.35×); microscopic view of the non-articular surface of the triquetral (1670 μm); microscopic view of the lateral surface of the pisiform (1670 μm). The bones show signs of bony breakdown on the non-articular surface (green oval), while the articular surfaces (blue arrows) appear majorly unchanged. The overall density of the carpals appears to be compromised. NOTE: The pisiform could not be photographed by the whole-specimen microscope.

**Figure 13:**
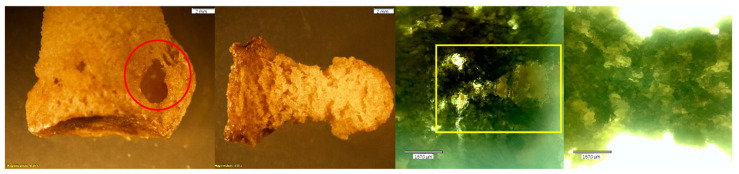
*Echo* and *Olympus* microscope photos showing bones after being submerged in HCl for 120 min. From left to right: macroscopic view of the dorsal distal portion of the PMP (2 mm; magnification 0.35×); macroscopic overall view of the DMP (2 mm; magnification 0.35×); microscopic view of the palmar distal portion of the PMP (1670 μm); microscopic view of the midshaft palmar view of the DMP (1670 μm). The greatest amount of change is visible in the bones after two hours. The PMP became hollow, with a strong, crystalline outer shell (red circle). The texture on both the PMP and DMP is indicative of how Hartnett et al. (2011) [9] described bone soaked in HCl: “the bone initially [became] porotic and pitted with eroded edges.” The internal structures look like melted plastic (yellow box) with newly formed holes running through the entire bone in areas where both the trabecular and cortical bone have been eaten away.

The micro- and macroscopic alterations before and after submersion in HCl (Figure 9; overall) are shown in photographs below to best illustrate the visible physical changes. In contrast to NaOH, the elements soaked in HCl showed changes within the first 30 min. The bony surface appeared crystalline and exhibited signs of cortical flaking and bone breakdown. The bone density appeared compromised after 1.5 h, with complete hollowing of the tubular bones after 2 h. All bones in HCl presented a ridge and furrow system due to chemical etching (Figure 10, Figure 11, Figure 12 and Figure 13). The HCl solution showed no change in pH throughout the experiment. Therefore, neither solution was neutralized within the two hours.

### 3.2. Dimensional Changes

**Figure 14:**
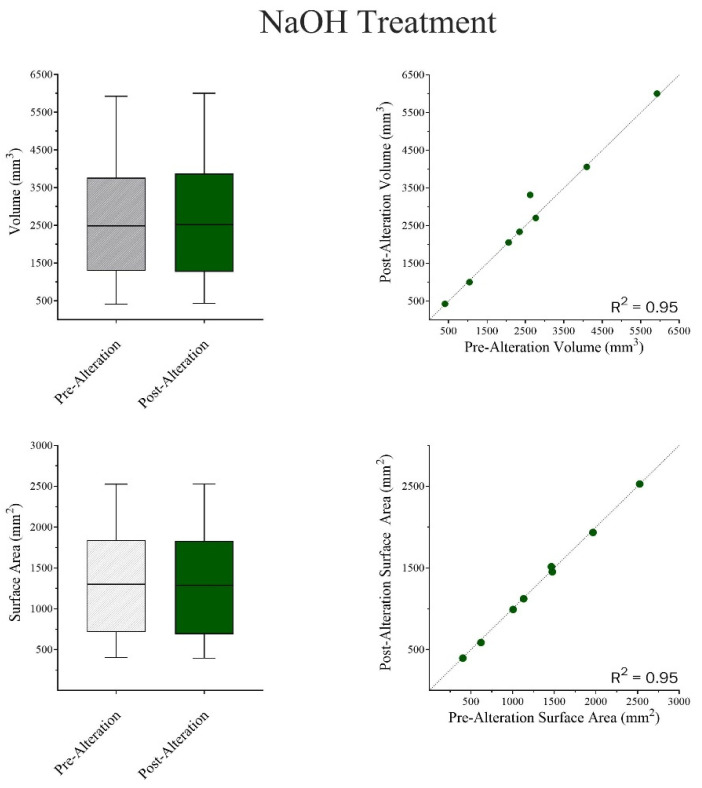
A Wilcoxon Matched Pair Signed Rank Test was used to compare pre- and post-alteration VOL (mm^3^) and SFA (mm^2^) in bone samples. Volume: W = 0.0; *p* > 0.999. The median difference (*Post-Pre*) = −8.6 mm^3^. Post-Alteration resulted in slightly smaller VOL compared to Pre-Alteration, but this finding is not statistically significant (**TOP**). Surface Area: W = −18.0; *p* = 0.250. The median difference (*Post-Pre*) = −12.3 mm^2^. Post-Alteration tends to have smaller SFA compared to Pre-Alteration. This suggests a decrease in SFA for most subjects, but this finding is not statistically significant (**BOTTOM**).

**Figure 15:**
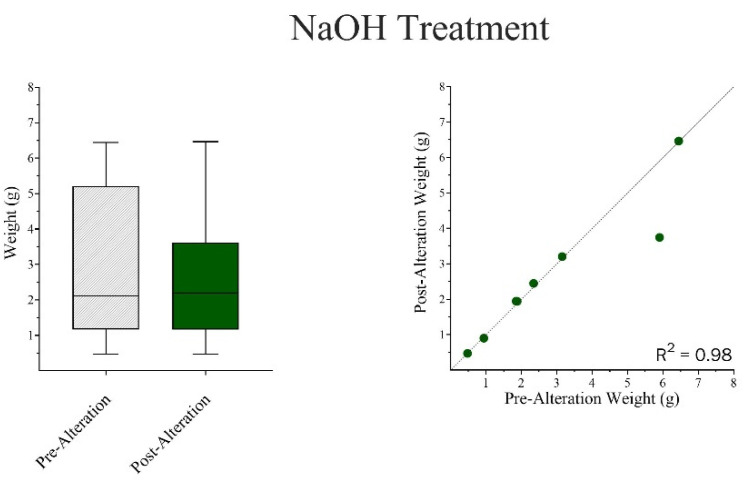
A Mann–Whitney U Test compared the changes in weight between the Pre-Alteration and Post-Alteration bone samples. There were no significant effects on weight; however, there may be a slight trend of increasing weight post-alteration due to the accumulation of by-products. Wilcoxon Matched Pair Signed Rank Test coincided with these results. Weight: W = 12.0; *p* = 0.4609. The median difference (*Post-Pre*) = 0.0360. The mean difference (*Post-Pre*) *=* −0.886 to 0.416 g.

**Figure 16:**
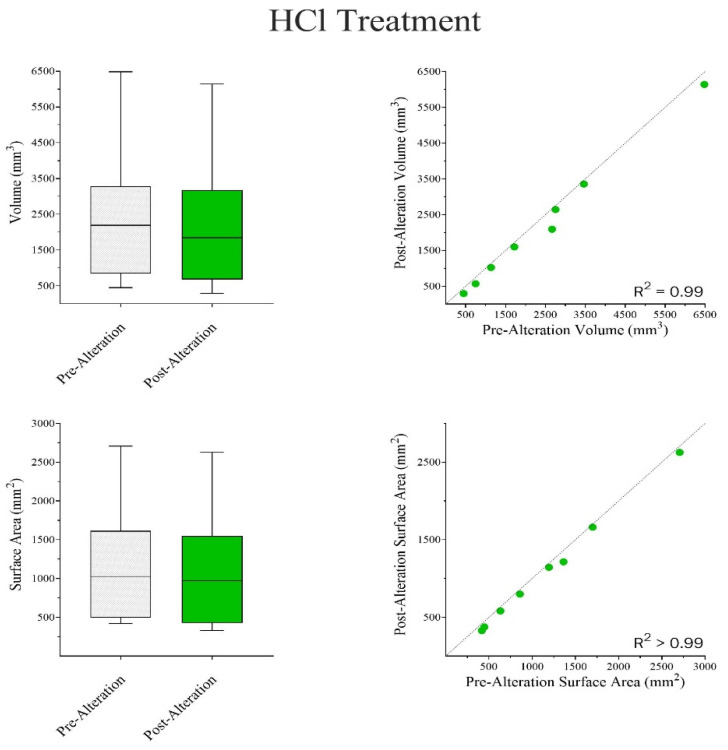
A Wilcoxon Matched Pair Signed Rank Test was used to compare pre- and post-alteration VOL (mm^3^) and SFA (mm^2^) in bone samples. Volume: W = −36.0; *p* = 0.008. The median difference (*Post-Pre*) = −139 mm^3^. The mean difference (*Post-Pre*) *=* −351.0 to −74.2 mm^3^. Overall, the Treatment led to a significant reduction in VOL (**TOP**). Surface Area: W = −36.0; *p* = 0.008. The median difference (*Post-Pre*) = −12.3 mm^2^. The mean difference (*Post-Pre*) *=* −103.0 to −44.6 mm^2^. Overall, there was a significant reduction in SFA Post-Alteration (**BOTTOM**).

**Figure 17:**
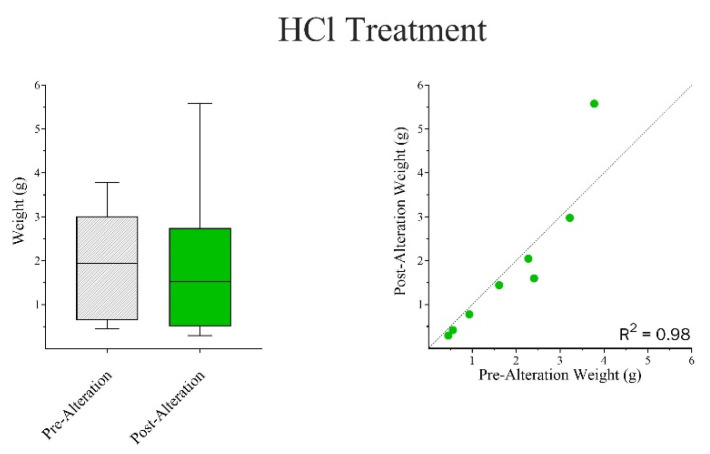
A Mann–Whitney U Test (Wilcoxon Rank Sum Test) was used to compare the changes in weight (grams) between the Pre-Alteration and Post-Alteration bone samples in HCl. While there was not a statistically significant effect on weight, there is an apparent reduction in weight observed. Wilcoxon Matched Pair Signed Rank Test coincided with these results. Weight: W = −20.0; *p* = 0.1953. The median difference (*Post-Pre*) =−0.164. The mean difference (*Post-Pre*) *=* −0.653 to 0.629 g.

Wilcoxon Matched-Pair Signed Rank and Mann–Whitney U tests were run independently for both chemical treatments. The results indicate that there were no significant differences in surface area-to-volume ratios between pre- and post-alteration bones in NaOH (Figure 14) but showed an apparent increase in weight (Figure 15). In the case of HCl, there were general reductions found in both surface area and volume measurements (Figure 16), as well as an apparent, but not statistically significant, reduction in weight (Figure 17).

### 3.3. DNA Analysis for Bone

The Nanodrop results indicate that the unaltered bone control produced 1.7 ng/μL of DNA, and the human-specific PowerQuant^®^ results show 0.0039 ng/μL. Evaluation of the Degradation Index (DI) indicated that all samples exhibited some degree of degradation, regardless of treatment, and IPC results showed no evidence of PCR inhibition in any sample.

**Figure 18:**
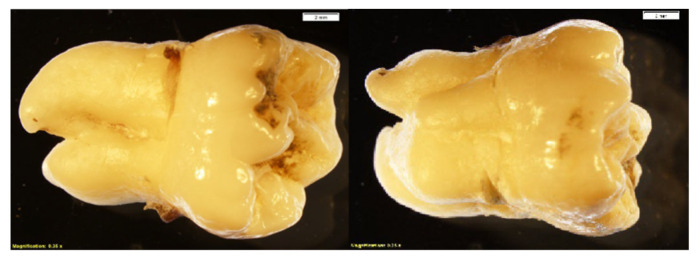
Unaltered control tooth (both sides shown) (2 mm; magnification 0.35×).

DNA yields were low, and most samples failed to generate STR profiles due to the limited quantity and reduced quality of the recovered DNA (Figure 17 and Figure 18). Only one incomplete autosomal STR profile (MC3; NaOH; 30 min) was obtained from the male sample, showing 20 of 23 loci, and none of the three Y-STR markers amplified. All remaining samples were processed for STR analysis but did not yield interpretable results, either because no alleles were detected or because all peaks fell below the analytical threshold of 150 RFU (Table 1).

### 3.4. Macroscopic Changes in Teeth

The molar used as the experimental control in phase 2 (Figure 18) endured no chemical alteration and exhibited no macroscopic changes.

**Figure 19:**
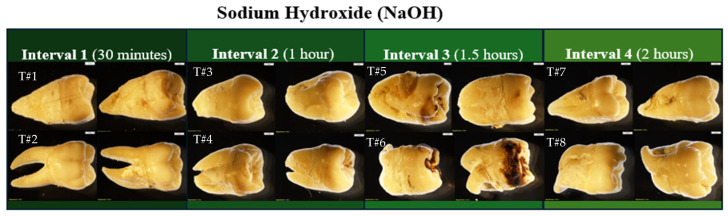
Olympus microscope photos of molars soaked in NaOH over the course of 2 h (2 mm; magnification 0.35×). Minimal to no change is observed throughout the experiment, but note the white coloration present throughout all intervals.

**Figure 20:**
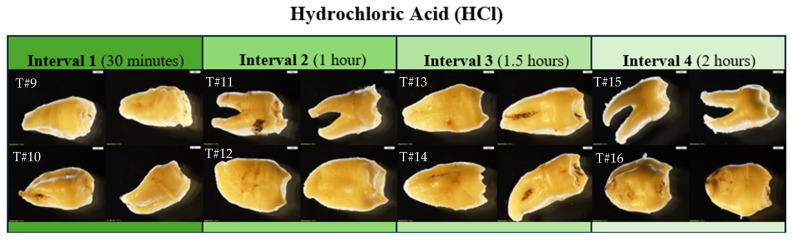
Olympus microscope photos of molars soaked in HCl over the course of 2 h (2 mm; magnification 0.35×). Alteration is visible within the first 30 min. Note the crenulation of the crowns throughout the experiment and cemental shedding of the roots, which is particularly visible after 2 h.

The teeth submerged in NaOH exhibited white coloration throughout the experiment but generally showed minimal to no morphological alterations (Figure 19). From the start, teeth placed in HCl showed morphological changes, beginning with the sharpening of the natural cusps and progressing to marked crenulation of the entire crown. Simultaneously, the roots exhibited an increasing rate of cemental shedding over time (Figure 20).

### 3.5. Dimensional Changes in Teeth

**Figure 21:**
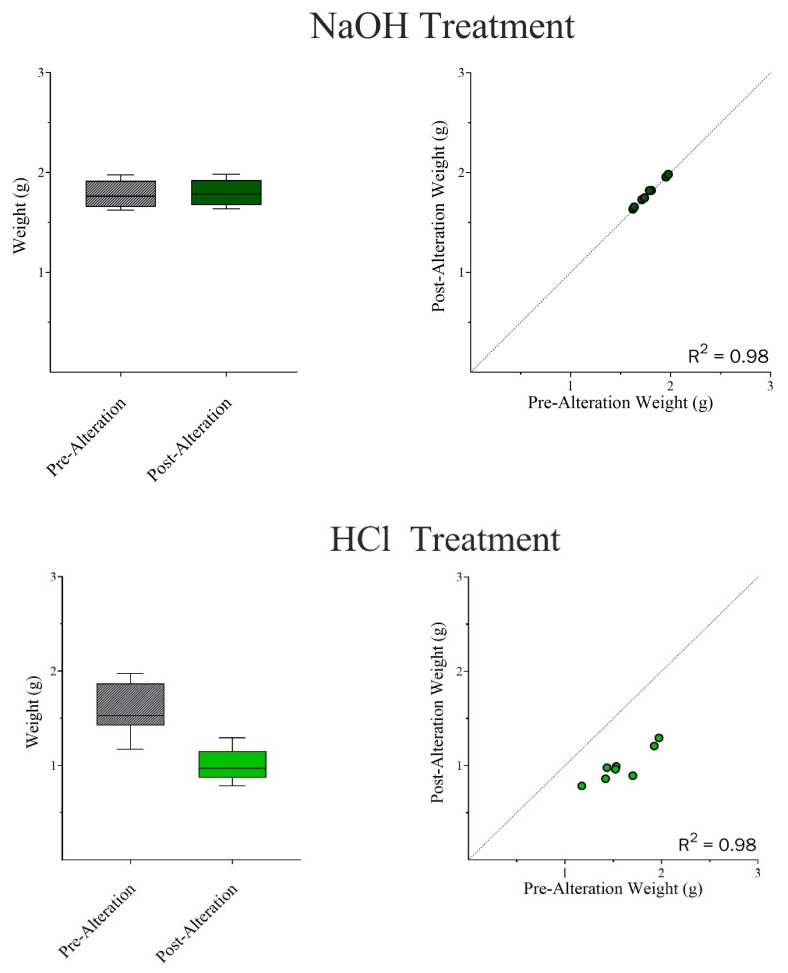
A Wilcoxon Matched Pair Signed Rank Test was used to compare the changes in weight (grams) between the Pre-Alteration and Post-Alteration tooth samples in both NaOH and HCl. Both the NaOH and HCl treatments were statistically significant given (*p* < 0.05). NaOH Weight: W= 36.0; P=0.0078. The median difference (*Post-Pre*) = 0.0140. The mean difference (*Post-Pre*) = 0.00692 to 0.00326 g (**TOP**). HCl Weight: W= −36.0; *p* = 0.0078. The median difference (*Post-Pre*) = −0.0561. The mean difference (*Post-Pre*) = −0.705 to −0.474 g (**BOTTOM**).

In phase two, only pre- and post-alteration weight data were collected for the tooth samples. The Wilcoxon Matched-Pairs Signed Rank test showed statistically significant differences between the Pre- and Post-alteration samples in both NaOH and HCl (Figure 21).

### 3.6. DNA Analysis for Teeth

The Nanodrop results indicate that the unaltered tooth control produced 41.90 ng/μL of DNA, and the pulp produced 169.80 ng/μL of DNA. PowerQuant^®^ results show 33.1604 ng/μL for the unaltered control dentin sample and 37.2498 ng/μL for the pulp sample. DNA yields were more affected by the NaOH treatment than by the HCl treatment. The DI for all but six teeth was below the threshold, and no inhibitors were detected in any sample, regardless of treatment. The DNA yield was sufficient to obtain complete STR profiles from all tooth samples (Table 2).

## 4. Discussion

### 4.1. Macroscopic and Dimensional Changes

Most chemical alteration studies focus on broad-scope changes leading to complete dissolution. Snedeker and colleagues’ pilot study, using porcine ribs, showed that elements placed in 100% crystal lye powder (1:1 ratio; 25 M) and 25–35% HCl, respectively, showed the most drastic alteration within the first 24 h of exposure [25]. In response to these findings and similar studies, the present study investigated intervallic changes to capture more robust, holistic data on what occurs to remains in the hours just after skeletonization (flesh removal). This is pertinent, as not every perpetrator will successfully take a body down to complete dissolution, given the time commitment and resources required.

Bone shape and volume were hypothesized to affect the alteration potential and the likelihood of DNA extraction. Specifically, higher volume bones were thought to resist chemical alteration better than lower volume bones. For example, round bones, like carpals, were hypothesized to yield more DNA due to their compact shape and lower surface area-to-volume ratio. Conversely, higher surface area-to-volume ratios should equate to greater chemical degradation of the long, thin shafts of tubular bones. However, this approach conflates morphological shape with bone density, which is more strongly influenced by the proportions and porosity of trabecular and cortical bone [3].

While some studies indicate that small trabecular-rich elements (i.e., carpals) or trabecular-rich epiphyses (i.e., metacarpals) are better sources of DNA compared to dense, tubular diaphyses, the reasons are not fully understood [3,14,15,16]. Leskovar and colleagues propose that DNA preservation may be more tightly linked to collagen denaturation, lower relative concentrations of phosphates, and lower crystallinity [15]. In combination, these studies justify the need for comprehensive micro- and macroscopic-level analyses, which, only in conjunction, can hope to answer the question of preservation potential on a case-by-case basis.

Though the elements soaked in NaOH showed little to no bone breakdown throughout, the elements in HCl, regardless of shape, were compromised by the environment. The densest portions of the carpals remained unchanged, while the non-articular surfaces and foramina showed signs of breakdown and eroded edges, respectively. The tubular bones showed the greatest changes, including alterations in surface texture and the breakdown of internal structures. By the end of the two-hour experiment, the greatest amount of change was visible in the HCl-treated bones, with the texture on many of the elements reflecting how Hartnett and colleagues (2011) described bone soaked in HCl: “the bone initially [became] porotic and pitted with eroded edges” [9]. It is reasonable to assume that these elements would follow a similar trajectory of softening, gelatinization, and movement toward amorphism. Future research should assess the rate of dissolution in fleshed remains under the conditions prescribed here.

Surface area and volume showed a strong negative correlation, with bone surface area (and thus attack area) increasing as volume decreased. There were no significant differences in surface area and volume ratios between pre- and post-alteration bones in NaOH. Still, there was an apparent increase in weight, possibly due to the formation of sodium salts. Conversely, there were general reductions in both measurements in remains placed in HCl, which also showed a non-statistically significant reduction in weight, given the necessarily small sample size. As expected, the relationship between these factors is not linear, and the results show a variable pattern of alteration that depends on the treatment and the duration of exposure. Neither NaOH nor HCl neutralized in either phase of the experiment but should be tested in longer time intervals to assess if there is a relationship between the rate of neutralization and DNA yield. A pH meter should be used in future experiments to avoid the issues of low resolution and subjectivity in color interpretation.

### 4.2. DNA Yields in Bone

It was expected that the amount of extractable DNA would decrease over time in both treatments [6,14,19,25], and that distinct micro- and macroscopic changes might indicate greater or worse DNA preservation. The NaOH treatment provided inconsistent results, making it difficult to draw a correlation between dimensional factors and DNA yield. In interval 1, the tubular bone (MC3) produced a higher DNA yield compared to its round counterpart (hamate). Interval 2 showed an inverse relationship, with the round element (trapezium), producing a higher yield compared to the tubular element (MC4). Though intervals 3 (scaphoid* and trapezoid) and 4 (PMP* and DMP) could not be compared for bone shape, both showed that elements with higher surface area and volume yielded greater amounts of DNA. Only two HCl-treated carpals (capitate and lunate) yielded DNA, and a direct comparison cannot be made because they are from separate intervals.

Neither a clear-cut trend of degradation/preservation over time, nor a direct comparison between macroscopic changes and concentrations could be observed, given that carpals and metacarpals have highly variable starting concentrations of DNA depending on postmortem conditions. This variability could have been exacerbated by the inability to separate bone layers during preparation and the differences in cortical and trabecular bone proportions between aliquoted extractions. As a result, the combination of low DNA concentrations and high DI prevented the retrieval of full or partial DNA profiles from the bones. Nonetheless, the DI indicated that differences are likely to exist between bones of varying morphological type (i.e., round vs. tubular) and may be better detected when replicating this experiment with larger sample sizes.

The STR results here contrast those found by Snedeker and colleagues (2024), who produced complete profiles from bone placed in NaOH for up to 4 weeks and from HCl up to 3 days after submersion [25]. It is possible that these samples produced greater yields due to bone type, initial soft tissue protection, and more rigorous decontamination prior to drilling. That said, the authors utilized a strict forensic extraction protocol, which involves a demineralization phase [26]. Comparing these results to a protocol designed for highly degraded (ancient) DNA would be particularly interesting for remains in HCl, which are already demineralized by the acid [27].

In the present study, the nondiscriminatory crushing of bone samples may have negatively impacted the quality of DNA sent for extraction and analysis. This type of mechanical stress could lead to further DNA fragmentation and possibly increase the risk of contamination. However, Steadman and colleagues (2006) suspect that the use of enzymes during maceration may negatively affect starting nDNA and contribute to limited DNA yields [21]; *Tergazyme* contains protease enzymes that target proteins rather than DNA.

Ultimately, while this study demonstrates promising DNA yields from small hand bones, it is important to acknowledge that these elements are susceptible to taphonomic alteration and are less frequently recovered in typical forensic contexts compared to larger bones, such as the femur. Nevertheless, in cases where hand bones are available, such as in well-preserved or partial remains, they may serve as a valuable supplementary source for genetic analysis.

### 4.3. Macroscopic Changes in Teeth

In phase two, the macroscopic changes to the teeth coincided with findings by Cope and Dupras (2009), in which HCl (31.45%) caused complete enamel loss and severe chemical etching of the molar crowns within the first hour [5]. It may be reasonable to assume that the teeth in the present study would follow a similar trajectory of increasing fragility and sponginess until complete gelatinization has occurred, but it is known that results tend to vary even when the same chemical concentrations are used.

Interestingly, even in cases where the powdered lye concentration differed from that used here, the teeth showed minimal to no macroscopic changes and exhibited a significant increase in mass, which the authors theorized may be due to liquid absorption or salt formation [5]. Teeth in HCl will likely maintain a recognizable morphology for at least half a day, showing chromatic changes on the surface and a progressive reduction in volume, but will reach dissolution before the 24 h mark [28].

### 4.4. DNA Yields in Teeth

DNA yields from teeth were more affected by the NaOH treatment than by the HCl treatment, likely due to the violent, exothermic reaction produced by the powdered lye and water mixture (Figure S1). Despite this, the degradation and inhibitor ratios were below the threshold, and the quantity was enough to obtain complete STR profiles from all tooth samples, regardless of treatment. The tooth samples outperformed the bone in this study, indicating better preservation potential. This is of utmost importance to forensic casework, as it demonstrates that, when possible, teeth should be selected for DNA sampling to achieve identificatory success even in difficult taphonomic contexts. Differences in tooth and bone anatomy best explain the discrepancies in DNA yields.

Enamel, which is more highly mineralized than bone, and cementum confer greater protection to the DNA-containing pulp and dentin [6,12]. Further, molars are considered excellent sources of genetic material due to their large pulp volume, multi-rooted structure, and overall greater proportion of DNA-containing cells; however, dentinal tubules, pulp chamber size, and pulp cellularity decrease both naturally due to age and in degradative environments [12]. Cementum may then become a more reliable source of DNA, due to its continuous deposition, as tooth structures become increasingly compromised [12,17].

In this study, cementum and enamel were removed, and DNA was obtained from dentin and pulp, which were well protected by the hard tissue layers, yielding high-quality DNA and full STR profiles. Though there is a range in DNA concentration between dentin and pulp within and between treatments, this, too, can be explained by differences between tissues within the dentin-pulp complex. Pulp is composed of blood vessels, nerves, and other cell types, which increase DNA yield, whereas dentin consists only of odontoblasts.

Moreover, some studies indicate that DNA yield varies depending on the area of the tooth sampled. The chemical treatment, as described in Castagnola et al. (2025) [24], demonstrated that tooth DNA is more affected by NaOH compared to HCl, regardless of tissue type. Irrespective of this, it was possible to obtain full STR profiles in most cases, demonstrating the effectiveness of this methodological approach as described previously in burnt remains [29] and chemical treatment [24] studies. While this protocol takes longer than the usual forensic procedure (pulverizing the entire tooth), the benefits of this workflow outweigh this disadvantage.

Future studies should not only assess if there is a correlation between the waning of exothermic temperature and DNA yield, given that high heat speeds up DNA degradation, but also compare sampling sites using a biology-centered approach.

### 4.5. Limitations of Chemical Alteration Studies

Small elements were used in this study out of necessity but evoke the need for informed sample selection in both experimental and forensic contexts. Elements of the hands and feet have been shown to be extremely susceptible to chemical alteration, often leading to the loss of recognizable morphological features and eventual full dissolution in both human ([9,30,31]; HCl) and animal proxy ([10]; NaOH) studies.

Udriştioiu and coauthors (2025) executed a critical review of the current state of the literature (17 articles involving human samples and 12 involving animal proxies), highlighting the overwhelming focus on total dissolution times and the categorization of chemicals by magnitude of alteration [8]. Issues with cross-validation arise because there is little overlap in concentrations, volumes, sample types, durations, and experimental foci. Even when the same treatment was used, the dissolution rate varied. These incongruent results are likely due to confounding factors, such as the application of substances. In the present study, the powder lye did not fully dissolve after warm water was added to the beakers. Therefore, even if all conditions had been identical to those in Snedeker et al.’s porcine pilot, our findings would likely have differed. Future iterations of this experiment should consider using a less concentrated NaOH solution (i.e., a higher water-to-powder ratio) and a magnetic stir bar to improve dissolution.

## 5. Conclusions

Forensic contexts bring about unique questions requiring scientific answers on a regular basis. This study took a comprehensive, intervallic look at the micro- and macroscopic changes in small bony elements and teeth treated with NaOH and HCl, respectively. Overall, HCl caused more marked visual changes in both bone and tooth samples, resulting in lower DNA yield in bone compared to teeth. Only one bone sample, Metacarpal 3 (MC3), produced a partial STR profile of 20 out of 23 autosomal markers. All teeth in HCl produced full STR profiles and yielded higher DNA concentrations compared to those in NaOH, which likely experienced more rapid denaturation due to the associated exothermic reaction. Despite this, NaOH showed no significant macroscopic changes in either the bone or tooth samples and produced full STR profiles. The bone samples in both treatments suggested apparent differences in weight pre- and post-alteration, but only general reductions in surface area and volume in HCl-treated samples. Finally, teeth in both treatments showed statistically significant changes in weight pre- and post-alteration. Overall, the trends in this study justify additional and expanded research. The approach taken here aims to guide chemical alteration studies toward more detailed experimental designs and away from myopically observing broad scope changes. Interdisciplinary partnerships will increase the feasibility of this type of scientific endeavor.

Given the paucity and contradictory nature of previous studies for comparison, and the necessarily small sample size utilized (i.e., low statistical power), this research design should be treated as a pilot study. Further research is required to determine whether cortical or trabecular bone is best for sample extraction, and to assess which skeletal elements have the highest DNA yields for analysis. Additionally, more knowledge is needed on the differential damage caused by chemicals at various volumes and concentrations so that the degree of compositional change may be compared to DNA preservation. Nevertheless, the general trends observed here provide justification for further analysis with larger sample sizes, longer durations, and more forensically significant conditions.

## Figures and Tables

**Table 1:** Bone samples according to treatment, treatment time, and DNA concentration measured by Nanodrop^®^ and human-specific quantification (PowerQuant^®^). The samples did not yield interpretable profiles, either because all peaks were below the analytical threshold of 150 RFU or because no detectable alleles were present. The only sample that produced a partial profile is MC3, indicated with an asterisk. Further details are provided in the text.

Bone Sample	Treatment	Time (min)	Nanodrop^®^DNA (ng/μL)	Powerquant^®^DNA (ng/μL)
(R) PMP	Control	0	1.70	0.0039
MC3 *	NaOH	30	2.20	0.0084
Hamate	NaOH	30	2.80	0.0011
Scaphoid	NaOH	90	1.10	0.0052
Ray 3 PMP	NaOH	120	1.50	0.0077
Ray 3 DMP	NaOH	120	2.60	0.0022
MC4	NaOH	60	2.10	0.0014
Trapezoid	NaOH	90	11.20	0.0008
Trapezium	NaOH	60	4.00	0.0031
MC5	HCl	30	3.20	0
Capitate	HCl	30	2.20	0.0002
Lunate	HCl	60	2.20	0.0012
MC2	HCl	60	6.50	0
Triquetral	HCl	90	1.50	0
Pisiform	HCl	90	0.00	0
Ray 1 PMP	HCl	120	0.00	0
Ray 2 DMP	HCl	120	0.00	0

**Table 2:** Tooth samples according to treatment, treatment time, and DNA concentration measured by Nanodrop^®^ and human-specific quantification (PowerQuant^®^). The DI for all but six teeth was below the threshold, and no inhibitors were detected in any sample, regardless of treatment . All samples yielded complete profiles. Further details are provided in the text.

Tooth Sample	Tissue Type	Treatment	Time (min)	Nanodrop^®^DNA (ng/μL)	Powerquant^®^DNA (ng/μL)
Control	Dentin	None	0	41.90	33.1604
Control	Pulp	None	0	169.80	37.2498
T #1	Dentin	NaOH	30	5.90	1.8218
T #2	Dentin	NaOH	30	11.30	0.5498
T #3	Dentin	NaOH	60	3.90	2.9541
T #4	Dentin	NaOH	60	2.80	0.3608
T #4	Pulp	NaOH	60	6.70	0.0117
T #5	Dentin	NaOH	90	0.80	0.149
T #5	Dentin	NaOH	90	1.00	0.132
T #6	Dentin	NaOH	90	2.30	1.1241
T #7	Dentin	NaOH	120	4.60	0.27
T #7	Pulp	NaOH	120	2.00	0.0435
T #8	Dentin	NaOH	120	7.60	5.7218
T #8	Pulp	NaOH	120	100.20	109.004
T #9	Dentin	HCl	30	33.40	23.8924
T #9	Pulp	HCl	30	72.40	42.4493
T #10	Dentin	HCl	30	37.00	26.5716
T #10	Pulp	HCl	30	68.90	40.1078
T #11	Dentin	HCl	60	18.30	11.9067
T #11	Pulp	HCl	60	30.00	15.7483
T #12	Dentin	HCl	60	19.40	16.7064
T #12	Pulp	HCl	60	118.50	73.3225
T #13	Dentin	HCl	90	27.50	33.9156
T #13	Pulp	HCl	90	56.90	47.1583
T #14	Dentin	HCl	90	5.10	3.4267
T #14	Pulp	HCl	90	0.10	0.0056
T #15	Dentin	HCl	120	1.00	0.9402
T #15	Pulp	HCl	120	74.30	46.7239
T #16	Dentin	HCl	120	11.10	7.6774
T #16	Pulp	HCl	120	82.20	30.8052

## Data Availability

The original contributions presented in this study are included in the article/Supplementary Material. Further inquiries can be directed to the corresponding authors.

## Supplementary Materials

The following supporting information can be downloaded at: https://www.mdpi.com/article/10.3390/biom16010001/s1, Figure S1: Surface temperatures taken every 15 min (max 120 min) over the first cup of NaOH with a non-contact handheld infrared (IR) thermometer.
