# Supplementary material for: Assessing the Micro- and Macroscopic Changes of Chemically Altered Human Bone and Teeth
Source: Biomolecules. 2025 Dec 19;16(1):1. doi: 10.3390/biom16010001 (PMC12838960; doi:10.3390/biom16010001)
Supplement: Supplementary file 1 [file biomolecules-16-00001-s001.zip › biomolecules-3974573-supplementary.pdf]

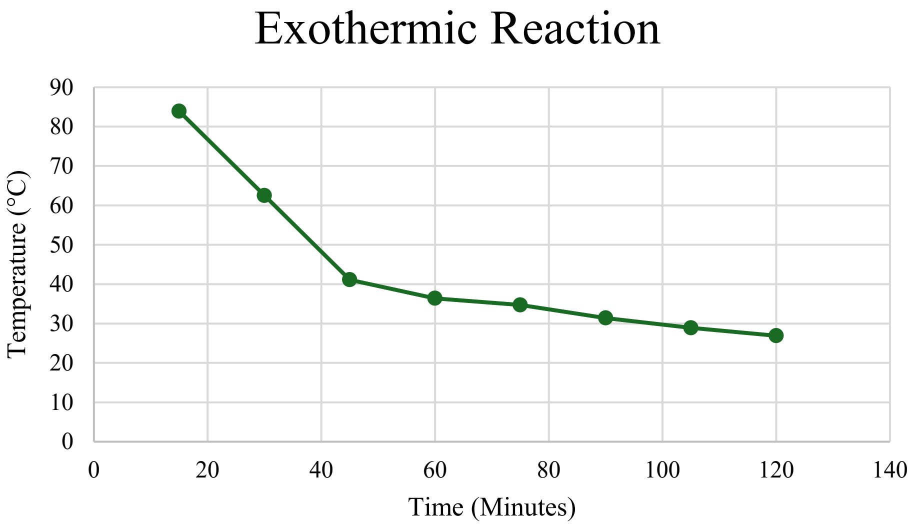

Figure S1. Surface temperatures taken every 15 minutes (max 120 minutes) over the first cup of NaOH with a non-contact handheld infrared (IR) thermometer.
